# Personalized prediction model for scar response after radionuclide therapy: development and validation in a Chinese cohort

**DOI:** 10.3389/fmed.2025.1655302

**Published:** 2025-10-13

**Authors:** Jinzhao Su, Jingbin Chen, Tianrong Wang, Tingwu Song, Haibin Xu, Shunshun Lin, Tiansheng Lin

**Affiliations:** ^1^Department of Nuclear Medicine, Fujian Medical University, Union Hospital, Fuzhou, China; ^2^Physiotherapy Department, Datian County General Hospital, Sanming, Fujian, China

**Keywords:** scars, prediction, image analysis, clinical features, web calculator

## Abstract

**Background:**

Scarring represents a persistent clinical and psychosocial challenge, with considerable variability in treatment response among patients. While both clinical and morphologic factors can influence outcomes, robust, individualized prediction of scar treatment efficacy remains elusive.

**Objective:**

To develop and validate an integrated predictive model for scar treatment outcomes using a combination of clinical and image-derived features in a Chinese cohort, and to translate this model into a web-based calculator for practical clinical application. This model requires validation in other ethnicities.

**Methods:**

We retrospectively analyzed 117 Chinese patients with scars treated at a single center, dividing them into a training (*n* = 83) and validation cohort (*n* = 34). Clinical data (including age, scar height) and quantitative features extracted from standardized scar photographs (solidity and mean saturation [S_mean]) were used to construct clinical, image-based, and combined predictive models. Feature selection was performed via LASSO regression, and models were developed using multivariate logistic regression. Model performance was evaluated using area under the receiver operating characteristic curve (AUC), calibration metrics (Brier score, log loss, HL test), and decision curve analysis (DCA). Net reclassification improvement (NRI) and integrated discrimination improvement (IDI) were calculated. A user-friendly web calculator was subsequently developed.

**Results:**

Scar height and age (clinical factors) as well as solidity and S_mean (image-derived metrics) were identified as independent predictors of poor treatment outcome. The combined model demonstrated superior discrimination (AUC 0.970 [training], 0.908 [test]), calibration, and clinical utility compared to clinical or image-based models alone. Calibration curves and metrics indicated excellent agreement between predicted and observed probabilities for the combined model. DCA, NRI, and IDI analyses further highlighted the incremental value and net benefit of the integrated approach. A web-based calculator was developed to enable individualized outcome prediction and support clinical decision-making.

**Conclusion:**

Integration of clinical and image-derived features enables robust, individualized prediction of scar treatment outcomes in this Chinese cohort. Our validated combined model, accessible via an easy-to-use web-based calculator, may enhance treatment planning, risk stratification, and patient counseling in scar management. Validation in diverse ethnic populations is essential.

## Introduction

Scarring represents a significant clinical challenge, affecting millions worldwide and leading to substantial physical, psychological, and social morbidity (1, 2). Scars can cause pain, itching, restricted movement, and disfigurement, impacting patients’ quality of life and self-esteem (3–5). Current treatment options for scars are diverse, ranging from topical agents and minimally invasive procedures to surgical interventions (6–8). However, the effectiveness of these treatments varies widely, and predicting individual patient response remains a major challenge (9–11). Clinical factors such as scar type, location, and patient age have been shown to influence treatment outcomes (5, 12), but these factors alone are often insufficient for accurate prediction (13).

The advent of radiomics and image analysis has opened new avenues for non-invasive assessment of tissue characteristics and prediction of treatment response in various medical fields (14–17). Image-derived features can capture subtle morphological and textural information that is not readily apparent on clinical examination, potentially providing valuable insights into the underlying biology of scars and their response to treatment (18, 19).

Despite the growing interest in radiomics, the application of image analysis to predict scar treatment outcomes remains limited (20). Few studies have explored the potential of integrating clinical and image-derived features to improve predictive accuracy. Furthermore, there is a lack of user-friendly tools to translate these predictive models into clinical practice.

Therefore, the primary objective of this study was to develop and validate a predictive model for scar treatment outcomes based on the integration of clinical and image-derived features in a Chinese cohort. We hypothesized that a combined model incorporating both types of data would provide superior predictive accuracy compared to models based on either clinical or image data alone. A secondary objective was to develop a web-based calculator to facilitate the clinical application of our predictive model. By addressing these objectives, we aim to improve treatment planning and patient counseling in the management of scars. However, the findings from this Chinese population require validation in other ethnicities to ensure broader applicability.

## Methods and patients

### Study design and patient population

This retrospective study included 117 Chinese patients treated for scars at Union Hospital, Fujian Medical University between 2020.01.01 and 2024.01.01. The study protocol was approved by the Union Hospital, Fujian Medical University Review Board (IRB) (protocol number: [2024-05-02]) and adhered to the principles of the Declaration of Helsinki. A waiver of informed consent was granted due to the retrospective nature of the study and the anonymized use of patient data.

**FIGURE 1:**
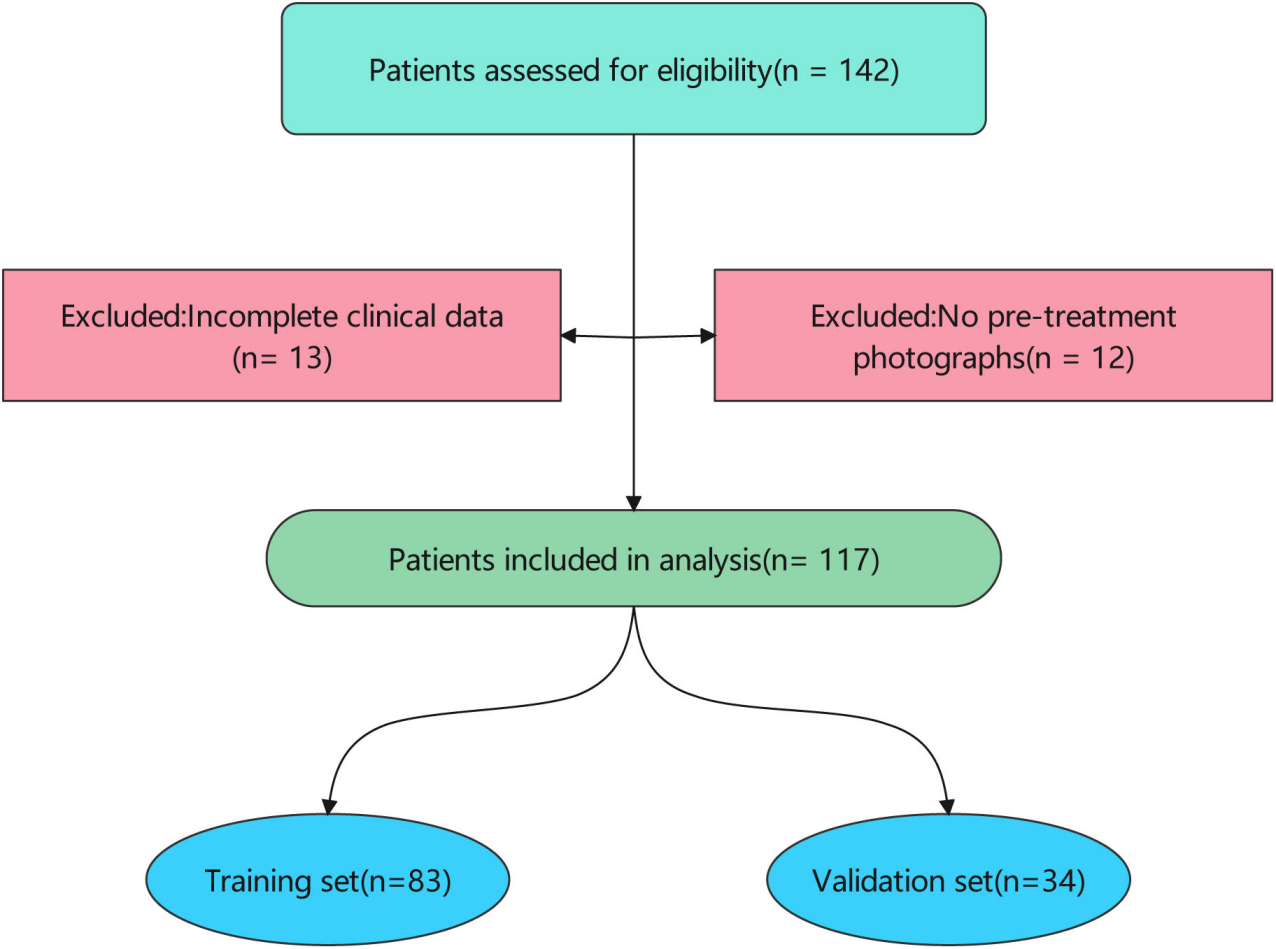
Patient inclusion flow. The patient cohort was randomly divided into a training set (n = 83) and a validation set (n = 34). Baseline characteristics were compared between the training and validation sets to ensure comparability.

Patients were eligible for inclusion if they had: (1) a clinical diagnosis of scar; (2) available clinical data, including age, sex, scar location, scar etiology, treatment course, and treatment dose; (3) standardized digital photographs of the scar region taken before treatment; and (4) documented treatment outcomes (Recovery vs. Scar) based on clinical assessment at 2025.01.01. Patients were excluded if they had incomplete clinical data or lacked pre-treatment photographs (Figure 1).

### Treatment protocol

Patients presenting to the outpatient clinic with scars underwent a comprehensive evaluation by a physician. Treatment decisions were individualized based on several factors, including patient age, scar duration, anatomical location, scar thickness, and prior treatment history. Based on these factors, a treatment dose ranging from 5 to 30 Gy was prescribed. The specific dose was selected based on the physician’s clinical judgment, with higher doses generally reserved for thicker, more recalcitrant scars.

Prior to treatment, all patients provided written informed consent after a thorough discussion of the risks and benefits of strontium-90 brachytherapy. Patients were then scheduled for treatment in the brachytherapy suite.

During treatment planning, a radiation therapist delineated the target volume, which encompassed the entire scar. For smaller scars requiring a single field, the surrounding normal skin was protected with adhesive tape. Larger scars were divided into multiple treatment fields to ensure uniform coverage.

Treatment was delivered using a strontium-90 ophthalmic applicator with a 2.5 cm × 2.5 cm active area. The applicator was placed in direct contact with the scar surface, and the prescribed dose was administered by controlling the application time. The reported dose represents the total dose delivered to each 2.5 cm × 2.5 cm treatment field.

### Outcome assessment

Scar treatment outcomes were assessed at 3, 6, and 12 months post-treatment by trained evaluators who were blinded to patient demographics, clinical data, and treatment parameters to reduce assessment bias. Treatment response was categorized as either “Recovery” or “Scar” based on a combination of standardized clinical and patient-reported outcomes. To standardize outcome assessment, the following validated scar assessment scales were utilized: Patient and Observer Scar Assessment Scale (POSAS) (21): The POSAS is a widely used scale that combines patient and observer ratings of scar characteristics, including pain, itching, color, thickness, and surface area.

### Definition of treatment outcomes

#### Recovery outcome

A reduction of at least 50% in the total POSAS score from baseline.

Patient satisfaction with the treatment outcome, as indicated by a score of ≤30.

Clinical assessment indicating significant improvement in scar appearance, including reduced thickness, improved color, and increased pliability.

#### Scar outcome

Failure to meet the criteria for a “Recovery Outcome.”

Worsening of scar symptoms (e.g., increased pain, itching).

Development of complications (e.g., ulceration, infection).

Patient dissatisfaction with the treatment outcome, as indicated by a score of >30.

### Clinical data collection

Clinical data were extracted from electronic medical records, including patient demographics (age, sex), height, weight, BMI, scar characteristics (location, etiology, length, width, height, duration), treatment parameters (course, dose), and treatment outcome (Recovery vs. Scar). Treatment outcome was defined as “Recovery” and “Scar.”

### Image acquisition and feature extraction

Standardized digital photographs of the scar region were acquired using a Canon EOS 70D digital camera with consistent lighting and magnification (1:1 macro lens, ISO 200, f/8 aperture). Images were preprocessed to ensure consistent orientation and scale using Adobe Photoshop. Regions of interest (ROIs) encompassing the scar area were manually delineated by a trained dermatologist blinded to treatment outcomes using ImageJ software (version 1.53k, National Institutes of Health, Bethesda, MD, USA). Image-derived features were extracted from the ROIs using Python with scikit-image library.

### Feature selection and model development

To identify the most predictive image-derived metrics, Least Absolute Shrinkage and Selection Operator (LASSO) regression was performed on the training set using R. The regularization parameter (λ) was selected using 10-fold cross-validation to minimize the cross-validation error.

Three predictive models were developed:

Clinical Model: Incorporating age and scar height.

Image Model: Incorporating Solidity and S_mean.

Combined Model: Incorporating age, scar height, Solidity, and S_mean.

Multivariate logistic regression was used to develop the clinical, image, and combined models. The models were trained on the training set and evaluated on the validation set.

### Model evaluation and validation

The performance of the models was evaluated using the area under the receiver operating characteristic curve (AUC), sensitivity, specificity, positive predictive value (PPV), negative predictive value (NPV), and accuracy. Calibration curves were generated to assess the reliability of the predicted probabilities. The Brier score, Log Loss, Hosmer-Lemeshow statistic, Mean Squared Error (MSE), and Mean Absolute Error (MAE) were calculated to quantify calibration performance.

Decision curve analysis (DCA) was performed to assess the clinical utility of the models. Net reclassification improvement (NRI) and integrated discrimination improvement (IDI) were calculated to quantify the incremental value of adding clinical or image information to the combined model.

### Web calculator development

A user-friendly web-based calculator was developed using Python with Streamlit to estimate the probability of treatment failure based on the combined model.

### Statistical analysis

Statistical analyses were performed using R4.1.3, and Python3.1.1. Continuous variables were presented as median [interquartile range (IQR)] and compared using Mann-Whitney U test. Categorical variables were presented as frequencies (percentages) and compared using chi-square test. A *p*-value < 0.05 was considered statistically significant.

## Result

### Basic information in all patients

This study included 117 Chinese patients, with a training set of 83 and a validation set of 34, to evaluate the efficacy of a treatment for scars. The majority of patients experienced recovery (69.2%), while 30.8% had persistent scars. The cohort was predominantly female (60.7%). Most scars were located on the trunk (76.9%), with a smaller proportion on the face (8.5%) and limbs (14.5%). The most common cause of scarring was unknown (76.9%), followed by operation (17.9%) and trauma (5.1%).

The median age of the patients was 27 years [IQR: 23, 35], with a median height of 1.64 meters [IQR: 1.58, 1.72] and a median weight of 56 kg [IQR: 50, 62], resulting in a median BMI of 20.32 kg/m^2^ [IQR: 19.23, 21.88]. The median treatment course was 3 sessions [IQR: 2, 5] with a median dose of 80 units [IQR: 60, 120]. The median scar length was 20 mm [IQR: 14, 35], the median scar width was 9 mm [IQR: 6, 12], and the median scar height was 2 mm [IQR: 1, 3]. The median duration of the scar was 60 months [IQR: 24, 120].

Statistical analysis revealed no significant differences between the training and validation sets for any of the baseline characteristics (all *p* > 0.05) (Table 1). This suggests that the two groups were well-matched and suitable for evaluating the treatment’s performance.

**TABLE 1:** All patients information.

Variable	Total	Training	Validation	Statistic	*P*_value
Recovery	81 (69.2%)	57 (68.7%)	24 (70.6%)	0	1
Scar	36 (30.8%)	26 (31.3%)	10 (29.4%)		
Female	71 (60.7%)	51 (61.4%)	20 (58.8%)	0	0.96
Male	46 (39.3%)	32 (38.6%)	14 (41.2%)		
Face	10 (8.5%)	6 (7.2%)	4 (11.8%)	1.15	0.56
Arm and leg	17 (14.5%)	11 (13.3%)	6 (17.6%)		
Strunk	90 (76.9%)	66 (79.5%)	24 (70.6%)	1.15	0.56
Trauma	6 (5.1%)	5 (6%)	1 (2.9%)	2.64	0.27
Operation	21 (17.9%)	12 (14.5%)	9 (26.5%)	2.64	0.27
Unknow	90 (76.9%)	66 (79.5%)	24 (70.6%)	2.64	0.27
Age (year)	27 [23, 35]	27 [23, 35]	28 [23, 34.75]	0.12	0.73
Height (m)	1.64 [1.58, 1.72]	1.65 [1.58, 1.72]	1.63 [1.58, 1.74]	0.26	0.61
Weight (kg)	56 [50, 62]	56 [50.5, 62]	56.5 [50.25, 65]	0.13	0.72
BMI	20.32 [19.23, 21.88]	20.15 [18.95, 21.95]	21.17 [19.68, 21.76]	0.95	0.33
Course	3 [2, 5]	3 [2, 4]	2.5 [2, 5]	0.09	0.77
Dose (Gy)	80 [60, 120]	85 [60, 120]	62.5 [60, 142.5]	0.27	0.6
Scarlong (mm)	20 [14, 35]	20 [13.5, 31]	27 [14, 39.25]	1.39	0.24
Scarshort (mm)	9 [6, 12]	9 [6, 12.5]	8 [5.25, 11.75]	0.41	0.52
Scarheight (mm)	2 [1, 3]	2 [2, 3]	2 [1, 3]	0.01	0.94
Scartime (month)	60 [24, 120]	48 [24, 114]	60 [19.5, 120]	0	0.96

### Information in the training set

**FIGURE 2:**
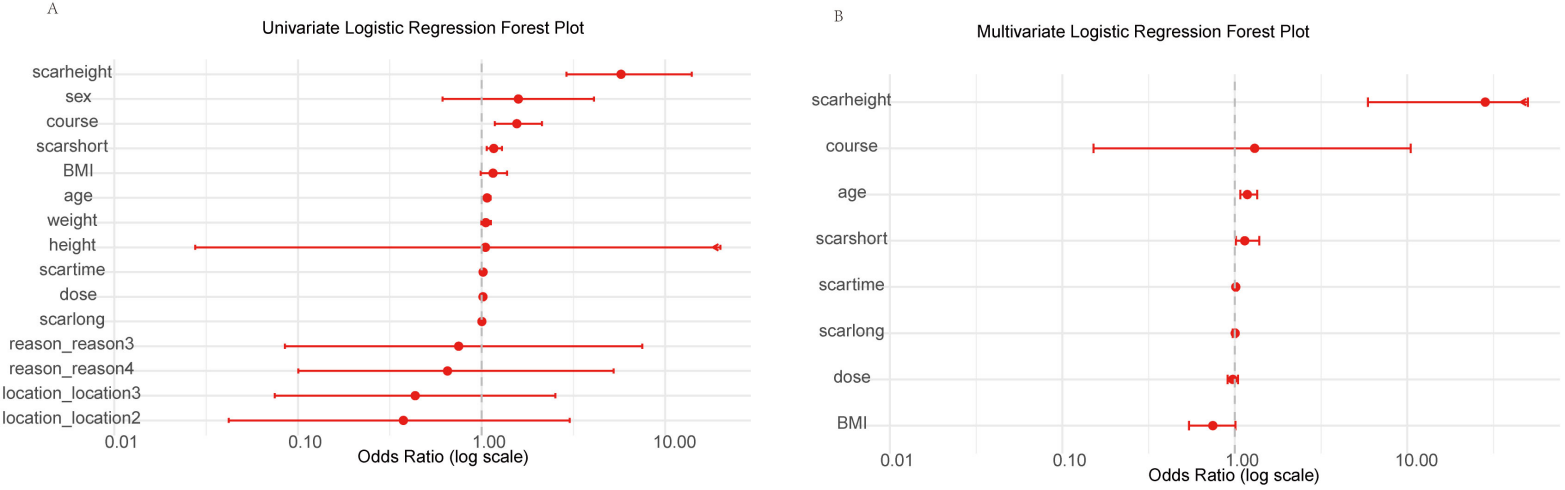
Forest plots of univariate and multivariate logistic regression analyses for clinical variables in the training set. **(A)** Univariate analysis showing odds ratios (OR) with 95% confidence intervals (CI) for each variable on a log scale (0.01 to 10.00), ordered by increasing OR. Arrows indicate CIs extending beyond axis limits. Scarheight demonstrates the strongest positive association with poor outcome. **(B)** Multivariate analysis for selected variables (*p* < 0.1 in univariate), with scarheight as the primary risk factor. Log-scaled *x*-axis (0.01 to 10.00); arrows for extended CIs.

To identify potential predictors of treatment outcome (defined as Recovery vs. Scar) in our Chinese cohort, we performed univariate analyses on a range of clinical variables within the training set (*n* = 83) using logistic regression to calculate odds ratios (OR), 95% confidence intervals (CI), and *p*-values for each variable. The results are visualized in a forest plot (Figure 2A). In the univariate analysis, scar height exhibited the strongest association with poor treatment outcome, with a high OR indicating increased risk of scar persistence. Other variables such as sex, course, scarshort, BMI, age, weight, height, scartime, dose, and scarlong also showed positive associations (OR > 1), suggesting potential predictive value. Categorical variables like location (levels 2 and 3), reason (levels 3 and 4) had ORs closer to or below 1, indicating weaker or protective effects. Notably, scar height, course, and scarshort appeared as prominent risk factors based on their distance from the null line (OR = 1).

To determine independent predictors, we performed multivariate logistic regression, including variables with *p* < 0.1 from univariate analysis (age, BMI, course, dose, scarlong, scarshort, scarheight, scartime). The results are shown in Figure 2B. After adjustment, scar height remained the dominant independent predictor with a markedly elevated OR, followed by course and age. Scarshort, scartime, scarlong, dose, and BMI had ORs nearer to 1 or wider CIs, reflecting reduced independent impact. Consistent with initial findings, only scar height (high OR, *p* = 0.001) and age (OR ≈ 1.18, *p* = 0.003) were statistically significant.

Based on these findings, we incorporated scar height and age into subsequent analyses to refine our predictive models and explore interactions with other variables.

### LASSO feature selection of image-derived metrics

**FIGURE 3:**
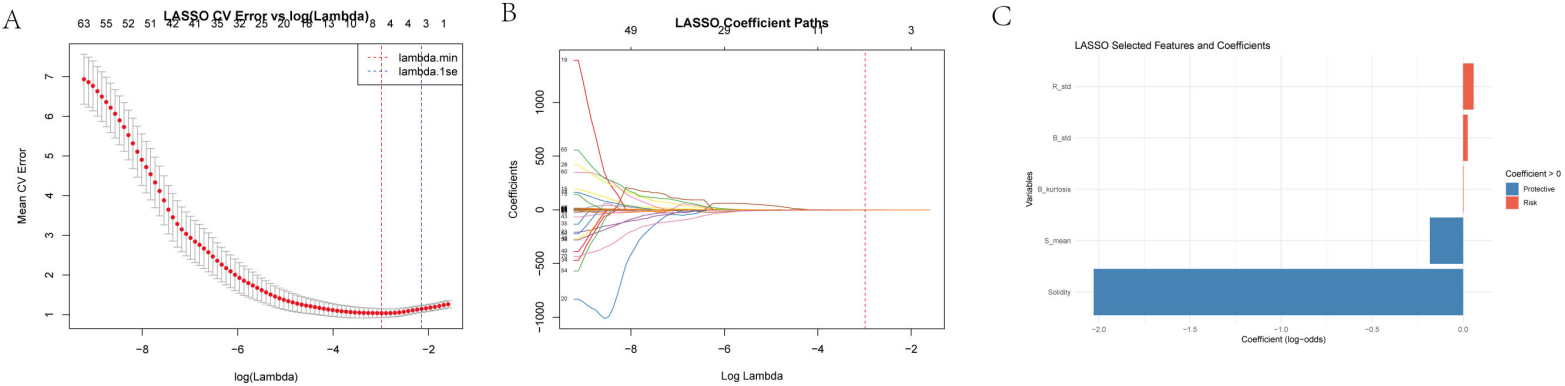
**(A)** LASSO cross-validation error vs. log(Lambda) this sub-figure depicts the relationship between the mean cross-validation (CV) error and the logarithm of the regularization parameter λ in the LASSO regression. The *x*-axis represents log(λ) ranging from – 8 to 7, and the *y*-axis shows the mean CV error. The minimum mean CV error occurs at λminλ*min*, and 1seλ is the largest λ within one standard error of the minimum error. This analysis helps in determining the optimal λ that balances model complexity and prediction error. **(B)** Log lambda vs. coefficients here, we present how the coefficients of the variables change as log(λ) varies. As log(λ) increases, the coefficients of many variables shrink toward zero. The number of non-zero coefficients decreases with increasing log(λ), demonstrating the variable selection property of the LASSO method. **(C)** Selected features and their clinical implications the LASSO regression selected “solidity” and “S_mean” as relevant image features for further analysis. Other features such as R_std (standard deviation of red - channel pixel values), B_std (standard deviation of blue - channel pixel values), and B_kurtosis (kurtosis of the blue - channel) were also considered in the initial analysis. R_std and B_std reflect the dispersion of red and blue intensities, respectively, and higher values may indicate an active or proliferative scar. B_kurtosis describes the sharpness of the blue - channel brightness distribution and can help identify abnormal scar patterns related to high - density tissue or calcification.

To identify the most predictive image-derived metrics for treatment outcome, we performed LASSO regression on features extracted from JPG images of the scar region (Figure 3). This analysis selected two key features: Solidity and S_mean (mean saturation).

Solidity, defined as the ratio of the scar area to its convex hull area, reflects the scar’s shape regularity and structural compactness. A higher solidity suggests a more regular and tightly packed scar structure. S_mean, representing the average color saturation, indicates the color concentration within the scar region. Lower saturation values suggest a more gray or white appearance, potentially indicative of reduced pigmentation and lower activity.

The LASSO model assigned a negative coefficient to Solidity and S_mean, suggesting it acts as a protective factor. This implies that scars with higher solidity and S_mean, indicating a more regular and compact structure, are associated with better treatment outcomes. These findings suggest that image analysis can provide valuable insights into scar characteristics that are predictive of treatment response. Solidity and S_mean, reflecting structural integrity and color maturity, respectively, may serve as useful imaging biomarkers for predicting treatment outcomes.

### SHAP bee-swarm

**FIGURE 4:**
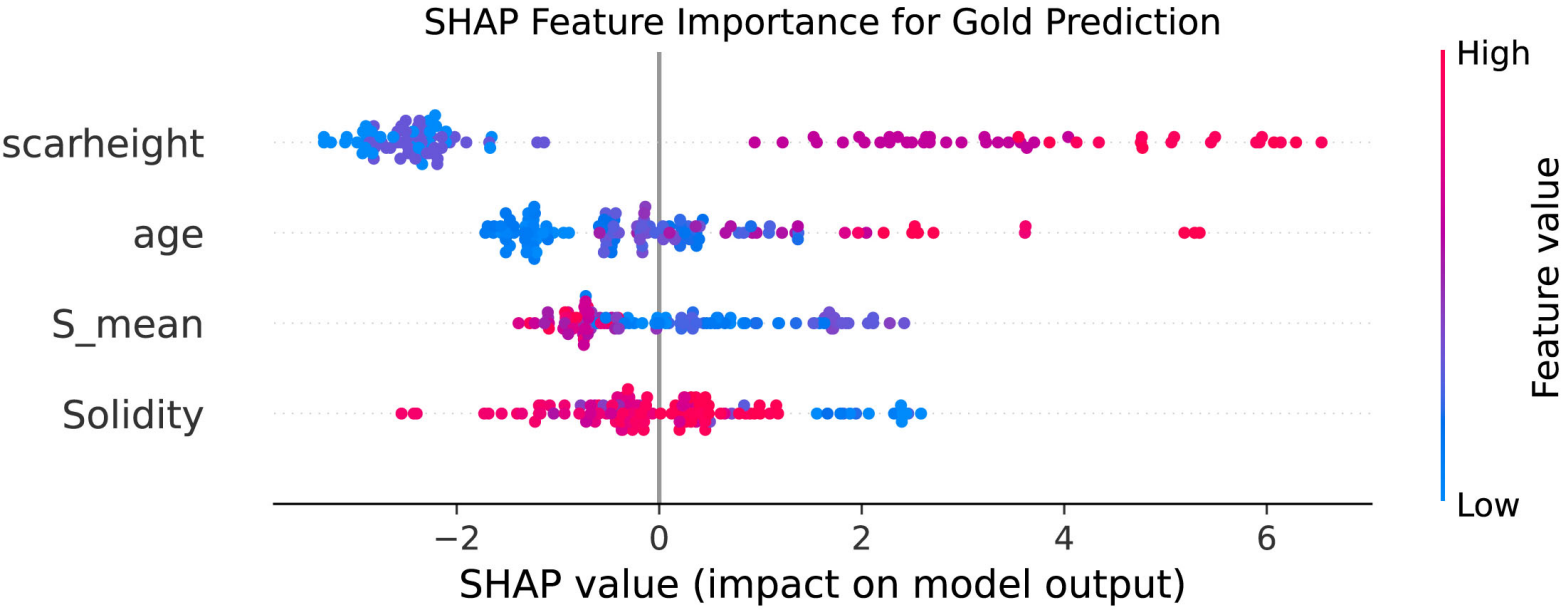
SHAP Summary plot of feature importance for prediction of scar outcome using random forest. The SHAP beeswarm plot ranks the top four predictors according to their overall impact on model output. Each point represents an individual case, with horizontal position denoting the SHAP value (the impact of the variable on the predicted outcome) and color indicating the feature value [from low (blue) to high (red)]. Solidity and S_mean show negative SHAP values for higher feature values, indicating protective effects against poor outcome. In contrast, higher scar height and age are associated with increased SHAP values, reflecting greater risk of poor prognosis. These findings confirm the importance and independent contributions of both image-based and clinical features in predicting scar treatment response.

To further validate the importance and directionality of key predictors, we applied a random forest model with SHAP (SHapley Additive exPlanations) analysis. The resulting SHAP summary (beeswarm) plot demonstrated that Solidity and S_mean were the influential protective features for predicting favorable treatment outcome, with higher values of these variables associated with lower risk of poor prognosis. Conversely, scar height and age were the top-ranking risk factors, as greater scar thickness and older age were linked to an increased probability of suboptimal response. The consistency of these results, in which both image-derived (Solidity and S_mean) and clinical (scar height and age) features dominate model performance, highlights their robust predictive value and complementary roles in assessing scar prognosis (Figure 4).

### Nomogram

**FIGURE 5:**
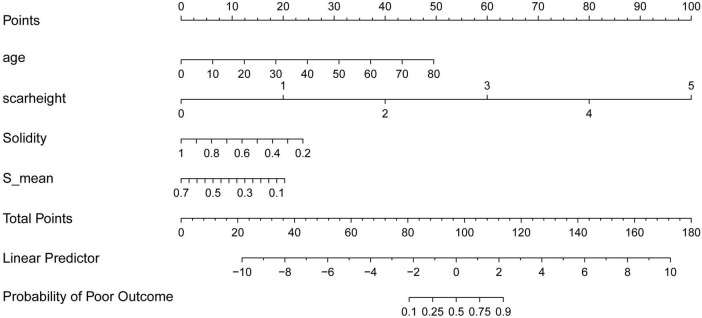
Nomogram for predicting the probability of poor treatment outcome. The nomogram incorporates age, scar height, solidity, and S_mean to predict the probability of a poor treatment outcome. To use the nomogram, assign points for each variable based on its value, sum the points to obtain a “total points” score, and then determine the corresponding probability of a poor outcome.

To facilitate clinical application of our findings, we constructed a nomogram (Figure 5) incorporating the four key predictors of treatment outcome identified by both LASSO regression and SHAP analysis: age, scar height, Solidity, and S_mean. The nomogram allows for individualized prediction of the probability of a poor outcome based on a patient’s specific values for these variables. To use the nomogram, a vertical line is drawn from each variable’s axis to the “Points” axis to determine the points assigned for that variable. The points for all four variables are then summed to obtain a “Total Points” score. Finally, a vertical line is drawn from the “Total Points” axis to the “Probability of Poor Outcome” axis to estimate the individual’s probability of a poor treatment outcome. The nomogram provides a user-friendly tool for clinicians to integrate these predictive factors into their decision-making process.

### ROC

**FIGURE 6:**
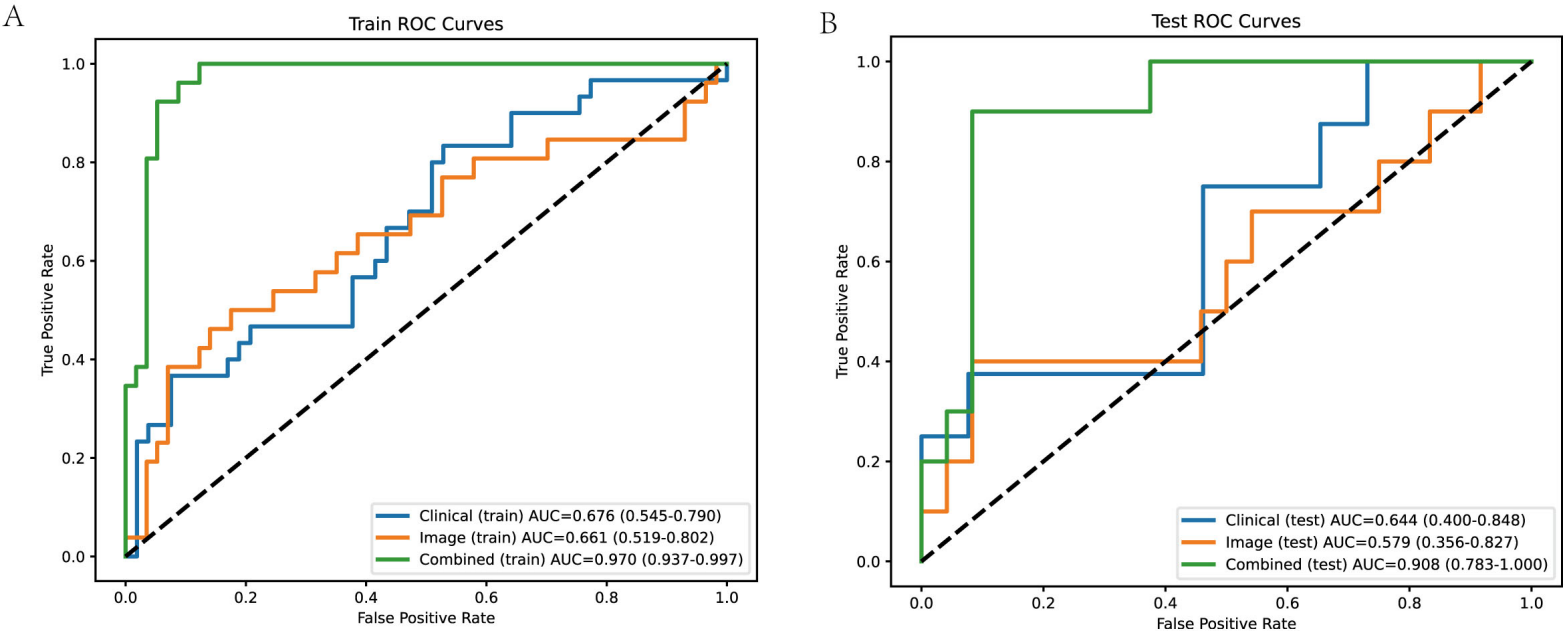
ROC curves of different models in training and test cohorts. **(A)** Training cohort ROC curves the combined model demonstrates the best performance, with an AUC of 0.970 (95% confidence interval: 0.937–0.997), indicating high accuracy in distinguishing between good and poor scar prognoses. The clinical model has an AUC of 0.676 (95% CI: 0.545–0.790), and the image model has an AUC of 0.661 (95% CI: 0.519–0.802). The AUC values are accompanied by their respective 95% confidence intervals, providing an indication of the reliability of the model performance. **(B)** Test cohort ROC curves the combined model still shows excellent performance, with an AUC of 0.908 (95% CI: 0.783–1.000). The clinical model has an AUC of 0.644 (95% CI: 0.400–0.848), and the image model has an AUC of 0.579 (95% CI: 0.356–0.827). The performance of the combined model in the test cohort indicates its good generalization ability, while the clinical and image models show relatively lower and less stable performance.

To evaluate the predictive performance of clinical, image-derived, and combined models, we constructed ROC curves for both the training and test cohorts (Figure 6). The clinical model, incorporating age and scar height, achieved an AUC of 0.676 (95% CI: 0.545–0.790) in the training cohort and 0.644 (95% CI: 0.400–0.848) in the test cohort. The image model, incorporating Solidity and S_mean, achieved an AUC of 0.661 (95% CI: 0.519–0.802) in the training cohort and 0.579 (95% CI: 0.356–0.827) in the test cohort. Notably, the combined model, incorporating all four variables (age, scar height, Solidity, and S_mean), demonstrated significantly improved performance, achieving an AUC of 0.970 (95% CI: 0.937–0.997) in the training cohort and 0.908 (95% CI: 0.783–1.000) in the test cohort. These results indicate that the combined model, integrating both clinical and image-derived information, provides superior predictive accuracy compared to models based on either clinical or image data alone. The sensitivity, specificity, PPV, NPV, and accuracy for each model in both cohorts are detailed in Table 2.

**TABLE 2:** Performance of different models in training and test cohort.

Model_group	AUC	AUC_CI_low	AUC_CI_high	Sensitivity	Specificity	PPV	NPV	Accuracy
Clinical_train	0.676	0.545	0.79	0.467	0.774	0.538	0.719	0.663
Clinical_test	0.644	0.4	0.848	0.375	0.654	0.25	0.773	0.588
Image_train	0.661	0.519	0.802	0.154	0.965	0.667	0.714	0.711
Image_test	0.579	0.356	0.827	0.4	0.917	0.667	0.786	0.765
Combined_train	0.97	0.937	0.997	0.769	0.965	0.909	0.902	0.904
Combined_test	0.908	0.783	1	0.9	0.917	0.818	0.957	0.912

The combined model shows superior performance across all metrics compared to clinical or image models alone, with notably higher AUC, sensitivity, and accuracy in both cohorts.

### Calibration

**FIGURE 7:**
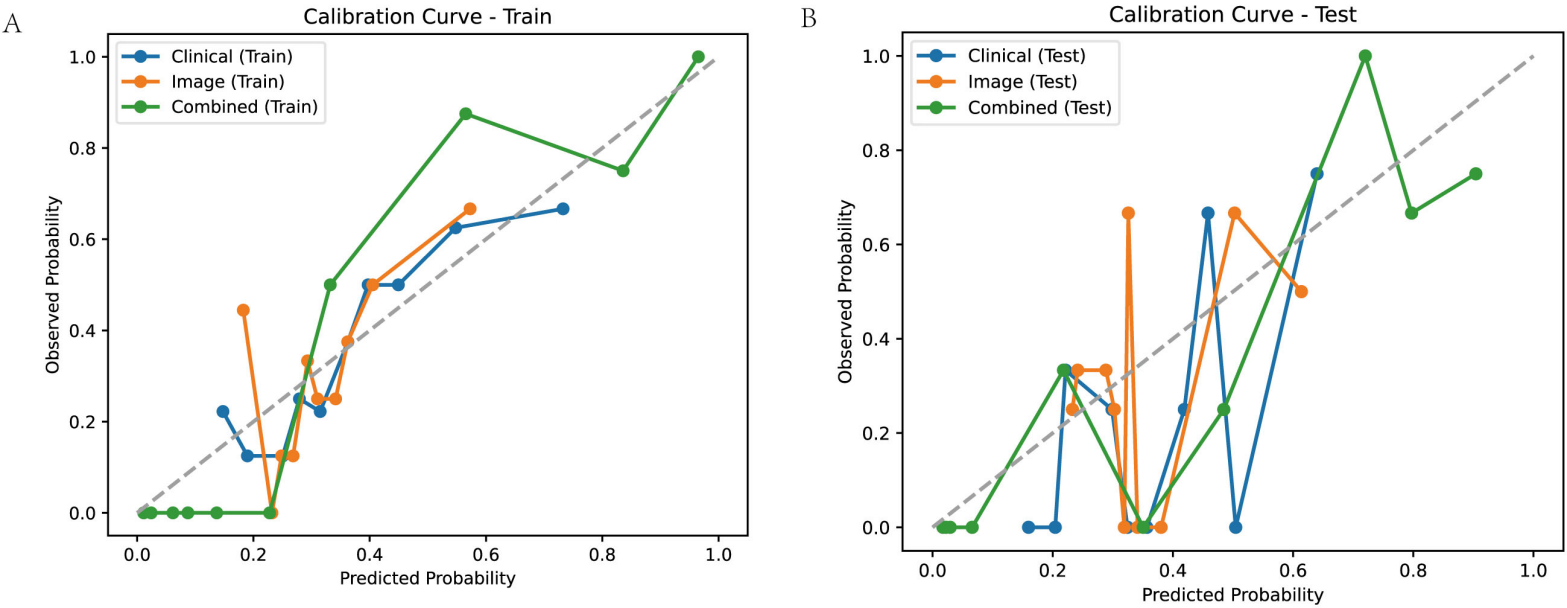
Calibration curves for predicting treatment outcome. Calibration curves for the clinical model, image model, and combined model in the training **(A)** and test **(B)** cohorts. Calibration curves plot observed probability versus predicted probability, with a perfectly calibrated model following the diagonal line.

To assess the reliability of the predicted probabilities generated by the clinical, image-derived, and combined models, we evaluated their calibration in both the training and test cohorts (Figure 7). Calibration curves plot the observed probability of a poor outcome against the predicted probability, with a perfectly calibrated model exhibiting a curve that closely follows the diagonal line. Visual inspection of the calibration curves revealed that the combined model exhibited better calibration compared to the clinical and image models, particularly in the training cohort. This suggests that the predicted probabilities generated by the combined model are more closely aligned with the actual observed probabilities.

To quantify the calibration performance, we calculated several metrics, including the Brier score, Log Loss, Hosmer-Lemeshow statistic (HL_Stat) and *p*-value (HL_*p*-value), Mean Squared Error (MSE), and Mean Absolute Error (MAE) (Table 3). The combined model consistently demonstrated lower Brier scores, Log Loss, MSE, and MAE compared to the clinical and image models, indicating improved calibration. The Hosmer-Lemeshow test revealed statistically significant miscalibration for the clinical and image models in both the training and test cohorts (HL_*p*-value < 0.01), suggesting that these models’ predicted probabilities deviate significantly from the observed outcomes. In contrast, the combined model did not show statistically significant miscalibration (HL_*p*-value > 0.05), further supporting its superior calibration performance. These results suggest that the combined model not only provides more accurate predictions but also generates more reliable probability estimates compared to the clinical and image models.

**TABLE 3:** Calibration performance of different model in training and test cohort.

Model	Dataset	Brier score	Log loss	HL_stat	HL_*p*-value	MSE	MAE
Clinical	Train	0.198	0.582	72.354	<0.01	0.198	0.400
Clinical	Test	0.163	0.505	84.241	<0.01	0.163	0.372
Image	Train	0.200	0.589	79.223	<0.01	0.200	0.405
Image	Test	0.193	0.576	65.189	<0.01	0.193	0.408
Combined	Train	0.075	0.156	10.27	0.246	0.075	0.181
Combined	Test	0.012	0.172	5.825	0.666	0.101	0.219

The combined model exhibits the best calibration, with lower brier scores, log loss, MSE, and MAE, and non-significant HL *p*-values, indicating reliable probability estimates.

### DCA

To further assess the clinical utility and added value of each model, we performed DCA and compared NRI and IDI metrics, with the combined model used as the reference.

**FIGURE 8:**
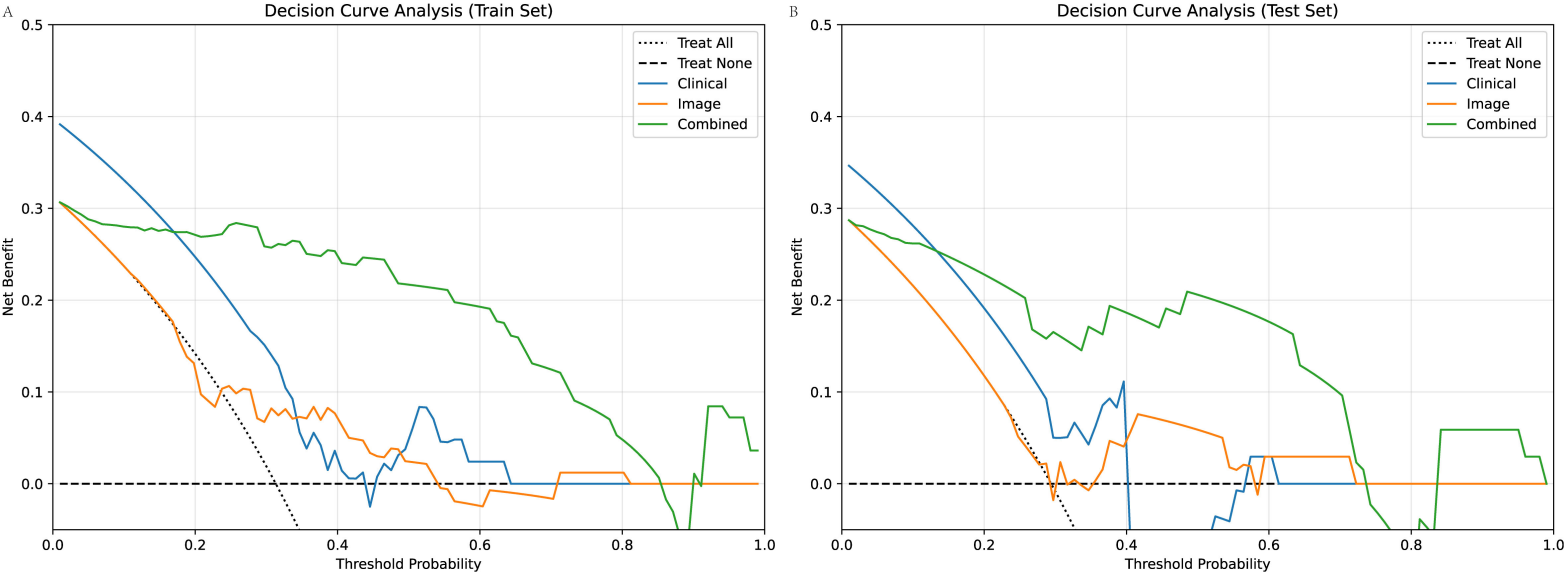
DCA of clinical, image-based, and combined models for predicting poor scar treatment outcomes. **(A)** DCA curves in the training cohort show that the combined model consistently yields the highest net benefit across a range of clinically relevant threshold probabilities compared to the clinical and image models**. (B)** Similar findings are observed in the test cohort, with the combined model offering superior clinical utility.

DCA curves (Figure 8) demonstrated that the combined model consistently provided the highest net benefit across a wide range of threshold probabilities compared to the clinical model and image-based model, in both training and test cohorts. This indicates that using the combined model for patient risk stratification can lead to improved decision-making and outcomes in real-world clinical practice.

Table 4 presents NRI and IDI values quantifying the incremental predictive value of the combined model over each single-domain model. Both the clinical and image-based models showed substantial positive NRI and IDI values when compared to the combined model in both the training and test sets (e.g., NRI for clinical: 0.388 in training, 0.558 in test; NRI for image: 0.58 in training, 0.416 in test; all IDIs > 0.46), reflecting significant improvements in both reclassification and discrimination when clinical and imaging features are integrated.

**TABLE 4:** RI and IDI of different model in training and test cohort (combined as reference).

Model	NRI (training)	IDI (training)	NRI (test)	IDI (test)
Clinical	0.388	0.516	0.558	0.471
Image	0.58	0.523	0.416	0.46

Collectively, these decision-analytic and reclassification measures provide robust evidence that the combined model offers greater clinical benefit and superior risk stratification performance versus models based on clinical or imaging features alone.

### Web calculator

To facilitate the clinical application of our predictive model and improve patient understanding, we developed a user-friendly web-based calculator, accessible at https://uuekzqcohkn65tbekanosn.streamlit.app/, that estimates the probability of treatment failure based on the four key predictors: age, scar height, Solidity, and S_mean. The calculator allows clinicians and patients to input individual values for these variables and obtain an immediate estimate of the probability of a poor outcome.

**FIGURE 9:**
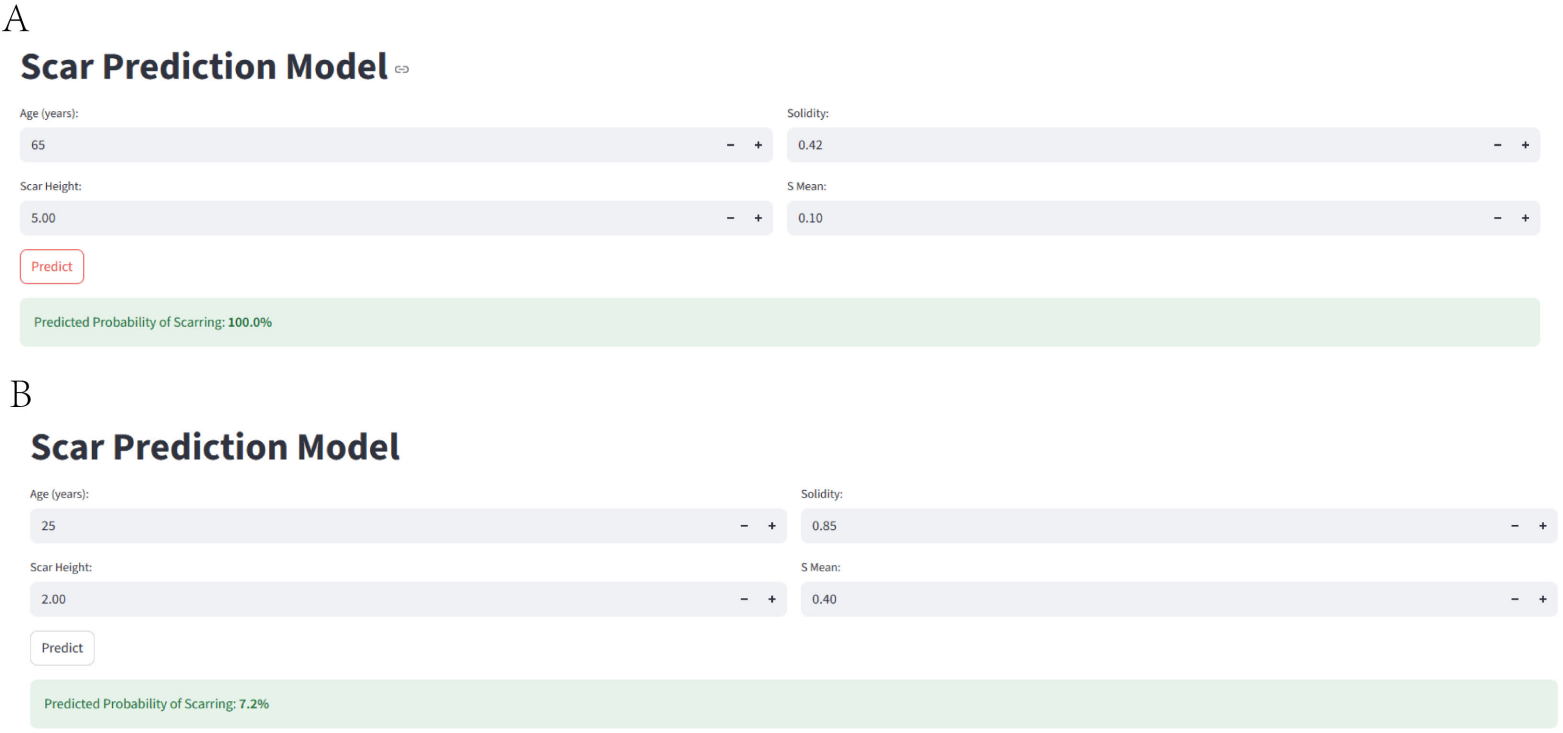
Web-based calculator for predicting treatment failure. The figure illustrates the web-based calculator developed to estimate the probability of treatment failure based on age, scar height, solidity, and S_mean. **(A)** Shows an example scenario with a high predicted probability of treatment failure, while **(B)** shows an example scenario with a low predicted probability of treatment failure.

**FIGURE 10:**
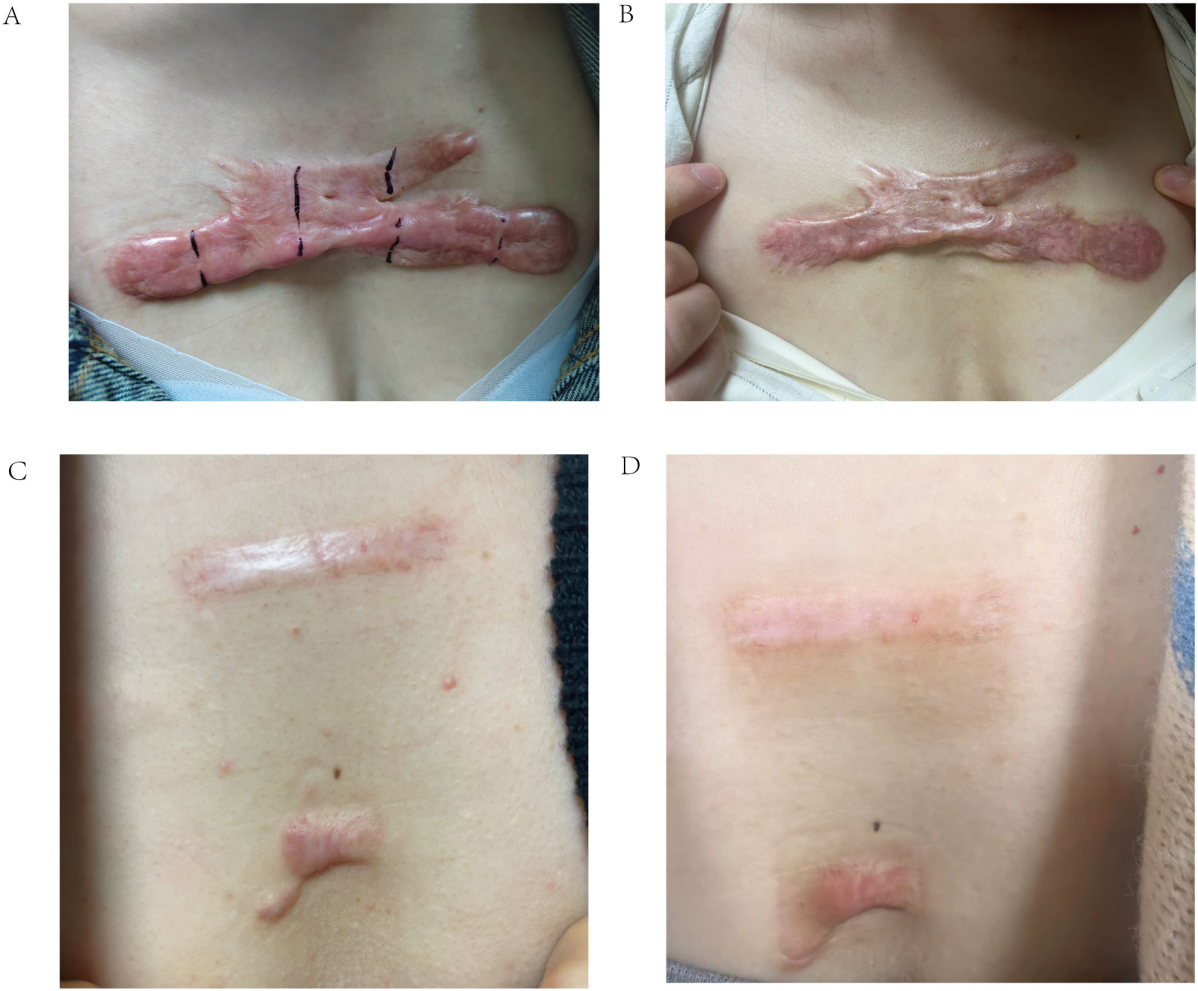
Representative clinical photographs of scar treatment outcomes. **(A)** Pre-treatment image of a 65-year-old female patient with a poor response to radionuclide therapy. **(B)** Post-treatment image of the same patient, demonstrating minimal improvement. **(C)** Pre-treatment image of a 25-year-old female patient with a good response to radionuclide therapy. **(D)** Post-treatment image of the same patient, showing significant scar reduction.

Figure 9 illustrates two example scenarios using the web calculator. In scenario A, a 65-year-old patient with a scar height of 5.00, Solidity of 0.42, and S_mean of 0.10 has a predicted probability of treatment failure of 100.0%. In contrast, in scenario B, a 25-year-old patient with a scar height of 2.00, Solidity of 0.85, and S_mean of 0.40 has a predicted probability of treatment failure of only 7.2%. These examples demonstrate the calculator’s ability to provide individualized risk assessments based on a patient’s specific characteristics, potentially aiding in treatment planning and patient counseling. Figure 10 presents the actual situations of these two patients.

## Discussion

This study demonstrates the potential of integrating clinical and image-derived features to predict treatment outcomes in Chinese patients with scars. Our findings highlight the independent predictive value of scar height and age (clinical factors) alongside Solidity and S_mean (image-derived metrics), and the superior performance of a combined model incorporating all four variables. The development and validation of a user-friendly web-based calculator further enhance the clinical translatability of our results.

Our findings demonstrate that scar thickness is an independent and robust predictor of treatment failure in radionuclide therapy. In our cohort, greater scar height was significantly associated with poor therapeutic response (OR 8.313, 95% CI 4.166–12.437, *p* = 0.001), consistent with the hypothesis that thicker scars impede uniform radiation penetration and reduce therapeutic efficacy. This observation aligns with prior studies indicating that reducing scar thickness through combined modalities—such as tension reducers and laser therapy—can significantly improve treatment outcomes. For example, Wang et al. reported that patients receiving combination therapy exhibited markedly reduced scar thickness compared to those receiving conventional treatment alone, correlating with superior clinical improvement (22). These findings underscore the clinical value of pre-treatment assessment and modulation of scar thickness to optimize therapeutic planning in scar management. Thicker scars may represent more established fibrosis and collagen deposition, potentially limiting the effectiveness of interventions.

Our study identified patient age as a significant factor influencing the response to radionuclide therapy in scar treatment. Specifically, older patients were more likely to exhibit poor therapeutic outcomes, with logistic regression analysis confirming age as an independent risk factor (OR 1.181, 95% CI 1.059–1.318, *p* = 0.003). This finding is consistent with previous evidence demonstrating age-related alterations in skin wound healing capacity and scar remodeling (23). In Wolle’s study, for each additional year of age, the odds of developing more severe scarring increased by 6.5% (95% CI: 5.8% to 7.2%) (24). The pathophysiological basis for this observation likely involves age-related changes in dermal structure and fibroblast function. Aging skin demonstrates reduced collagen turnover, diminished fibroblast proliferation, and impaired neovascularization—all of which are essential for effective scar remodeling and response to localized radionuclide-induced cytotoxicity. Moreover, older individuals tend to exhibit increased oxidative stress and altered inflammatory responses, which may hinder the reparative processes required following radionuclide exposure. These findings echo clinical observations in other scar treatment modalities. For instance, studies have shown that younger patients respond more favorably to laser therapy and surgical scar revision, likely due to their enhanced regenerative capacity and more dynamic dermal cellular activity (22). Thus, patient age should be considered a critical factor in pre-treatment assessment and personalized therapeutic planning for radionuclide-based scar interventions.

Our analysis identified two key radiomic features—Solidity and S_mean—as significant predictors of treatment response in radionuclide-based scar therapy. In our cohort, lower Solidity was independently associated with poor therapeutic outcome, suggesting that irregular and infiltrative scar margins might reflect a more fibrotic or biologically active phenotype less responsive to localized radionuclide damage. This aligns with previous radiomic studies in oncologic imaging, where reduced solidity often correlates with infiltrative or aggressive tissue behavior (25). Similarly, S_mean, a texture-derived radiomic feature reflecting the average signal intensity in grayscale, showed a strong inverse association with response, with lower values predicting treatment failure. Prior research by Lu et al. demonstrated that reduced signal intensity heterogeneity may be linked to denser collagen deposition and reduced vascularity, factors that could impair radionuclide diffusion and local cytotoxicity (26). Notably, while their study focused on hepatic fibrosis imaging, the mechanistic parallels in extracellular matrix remodeling provide biologically plausible support for our findings. Interestingly, although other studies in radiomic modeling have found high S_mean values associated with poor prognosis in hypervascular tumors (e.g., gliomas) (27), such trends were not replicated in our scar-based dataset. This discrepancy likely stems from fundamental differences in tissue composition: unlike tumors, fibrotic scars lack neovascular proliferation and exhibit low metabolic activity, leading to different radiomic signal patterns. Taken together, our results highlight that Solidity and S_mean capture biologically relevant microstructural and compositional features that significantly influence therapeutic response, and may serve as non-invasive imaging biomarkers for personalized treatment stratification.

This study demonstrates the potential of combining clinical and image-derived features to predict scar treatment outcomes, offering a personalized approach to radionuclide therapy (28). Our findings align with the growing body of evidence supporting the use of radiomics in predicting treatment response across various medical domains (29). For instance, a study by Ma et al. found that image features extracted using self-supervised learning showed promising internal prediction performance for local control, regional control, and distant metastasis-free survival in oropharyngeal cancer patients (30). Similarly, Zhou et al. demonstrated that radiomics features from (18)F-FDG PET scans have potential added value to clinical features in predicting treatment response and prognosis in diffuse large B-cell lymphoma patients (31). In our study, the combined model’s high AUC (0.970 in training, 0.908 in validation) indicates excellent discriminative ability, meaning it can reliably distinguish between patients likely to achieve recovery and those at risk of persistent scarring, which is crucial for tailoring treatment plans. The low Brier score (0.075 in training, 0.012 in validation) reflects strong calibration, ensuring that predicted probabilities closely match actual outcomes and thus provide trustworthy risk estimates for patient counseling. Furthermore, the substantial NRI values (e.g., 0.388–0.58 in training) signify that the combined model reclassifies a significant proportion of patients more accurately than single-domain models; for example, an NRI of 0.55 implies that approximately 55% of patients are correctly shifted to higher or lower risk categories, potentially guiding decisions on whether to pursue aggressive therapies or conservative management. Similarly, the IDI values (> 0.46) demonstrate improved discrimination, enhancing the model’s ability to separate risk groups and supporting better-informed clinical choices in real-world settings, such as prioritizing high-risk patients for adjunctive interventions.

Furthermore, our study highlights the importance of feature selection in radiomics modeling. We employed LASSO regression to identify the most relevant image-derived metrics, while other studies have used different feature selection methods, such as random forest. The choice of feature selection method can significantly impact model performance and generalizability (31). However, our image analysis was limited to 2D photographs, which may not fully capture the three-dimensional complexity and subsurface characteristics of scar tissue. Advanced imaging modalities, such as 3D imaging techniques (e.g., stereophotogrammetry), optical coherence tomography (OCT), or confocal microscopy, could provide volumetric data, microstructural details (e.g., collagen fiber organization and depth of fibrosis), and vascularity assessments that are not visible in standard 2D images. Integrating these modalities into future models may further improve prediction accuracy by incorporating additional quantitative features, such as scar depth profiling or real-time tissue perfusion metrics, ultimately leading to more precise individualized predictions.

The development of a web-based calculator provides a practical tool for clinicians to implement our findings in their daily practice. By simply inputting a patient’s age, scar height, Solidity, and S_mean, clinicians can obtain an individualized estimate of the probability of treatment failure, potentially aiding in treatment planning and patient counseling. The calculator also empowers patients to better understand their individual risk and participate more actively in treatment decisions.

### Limitations

This study has several limitations that should be acknowledged. First, the retrospective design may be subject to selection bias and data completeness issues, potentially influencing the reliability of the findings. Second, the sample size, while adequate for the analyses performed, is relatively small (*n* = 117), which may limit the statistical power and generalizability of the results. Third, the study was conducted at a single center in a Chinese population, which may further restrict applicability to other ethnicities and healthcare settings; external validation in larger, multicenter, and prospective cohorts is required to address these issues and confirm the model’s robustness. Fourth, the definition of “Recovery” and “Scar” was based on clinical assessment, which may be subjective. Future studies should incorporate objective measures of scar improvement, such as Vancouver Scar Scale, Cutometer, skin ultrasound. Fifth, the image analysis was performed on 2D photographs, which may not fully capture the complexity of scar tissue. Future studies should explore the use of 3D imaging techniques, such as optical coherence tomography, confocal microscopy. Finally, the web-based calculator has not yet been formally validated in an independent cohort. Additionally, while our findings demonstrate predictive value for the identified variables in radionuclide therapy for scars, these must be validated in other cohorts to confirm their broader applicability.

### Future directions

Future research should focus on validating our findings in larger, multi-center prospective studies, including diverse ethnic populations. Further investigation is needed to explore the biological mechanisms underlying the associations between Solidity, S_mean, and treatment outcome. Studies are also needed to evaluate the cost-effectiveness of using the combined model and web-based calculator in clinical practice. Additionally, future research should explore the potential of incorporating other clinical and image-derived features into the predictive model, such as scar vascularity, collagen organization.

## Conclusion

These findings have the potential to improve treatment planning and patient counseling in the management of scars. However, validation in other ethnicities, as well as in larger, multicenter, and prospective cohorts, is necessary to confirm generalizability and enhance the model’s clinical utility.

## Data Availability

The raw data supporting the conclusions of this article will be made available by the authors, without undue reservation.
